# Health care trajectories and barriers to treatment for patients with end-stage renal disease without health insurance in Mexico: a mixed methods approach

**DOI:** 10.1186/s12939-020-01205-4

**Published:** 2020-06-08

**Authors:** Marcela Agudelo-Botero, María Cecilia González-Robledo, Hortensia Reyes-Morales, Liliana Giraldo-Rodríguez, Mario Rojas-Russell, Dolores Mino-León, Dayan Irene Ocampo-Morales, Rafael Valdez-Ortiz

**Affiliations:** 1 Politics, Population and Health Research Center, School of Medicine, National Autonomous University of Mexico, Mexico City, Mexico; 2 Center for Health Systems Research, National Institute of Public Health, Cuernavaca, Morelos Mexico; 3 Department of Demographic Epidemiology and Social Determinants, National Institute of Geriatrics, Mexico City, Mexico; 4 Faculty of Higher Studies Zaragoza, National Autonomous University of Mexico, Mexico City, Mexico; 5 Research Unit in Clinical Epidemiology. Specialty Hospital of the 21st Century National Medical Center, Mexican Institute of Social Security, Mexico City, Mexico; 6 Nephrology Services, General Hospital of Mexico “Dr. Eduardo Liceaga”, Mexico City, Mexico

**Keywords:** Chronic kidney disease, Health care, Barriers, Renal replacement therapy

## Abstract

**Background:**

Mexico has the sixth-highest premature death rate from chronic kidney disease (CKD) in the world. From 1990 to 2017, the age-standardized CKD mortality rate jumped from 28.7 to 58.1 per 100,000 inhabitants, making it the second-leading cause of death that year. Medical care for the disease is inequitable, as those without health insurance have limited access to renal replacement therapy (RRT). The objective of this study is to describe the healthcare trajectories of patients with end-stage renal disease (ESRD) in a public hospital in Mexico City and the barriers they face in receiving peritoneal dialysis and haemodialysis.

**Methods:**

This study uses a convergent mixed methods approach and is predominantly qualitative. Patients completed 199 surveys, and 42 semi-structured interviews with patients having ESRD and their families were conducted. The quantitative data were analysed using descriptive statistics, and the qualitative data were processed using a phenomenological approach.

**Results:**

It was found that 76.9% of the patients received peritoneal dialysis or haemodialysis as their first RRT. Over 30% began their treatment at least a month after a health professional prescribed it. Almost 50% had been hospitalized for complications related to the disease in the previous year, and 36% had uncertainties about their treatment. Close to 64% of the haemodialysis patients received treatment intermittently. Barriers to accessing treatment, information, contact with health services, and treatment availability were identified. Patients and their families encountered economic and emotional difficulties at every phase of their search for medical care and treatment.

**Conclusion:**

Mexico urgently needs to implement public policies related to CKD that are primarily directed at its prevention but should also implement policies directed at slowing its progression, reducing its complications, and providing funding for uninsured patients who require RRT. These policies must be based on the perspectives of human rights and equality, and the perspectives of patients, their families and the general population should be included in the policy creation process.

## Background

Chronic kidney disease (CKD) is currently a rapidly rising public health problem [1–4]. It is estimated that in 2017, there were 697.5 million cases globally of all-stage CKD, with a prevalence of 9.1%. That same year, close to 1.2 million people died of this disease [1].

Mexico has the world’s sixth-highest premature death rate from CKD [1]. From 1990 to 2017, the country’s age-standardized CKD mortality rate jumped from 28.7 to 58.1 per 100,000 inhabitants [1, 5], thus making this disease the second leading cause of death that year. This jump represented an increase in the mortality rate of 102.3% [5], while the increases in the rates of Latin America and the worldwide population were 32.9 and 2.8%, respectively [1, 6]. This remarkable increase in the mortality rate is explained by the following factors: 1) a growing burden of risk factors such as diabetes, hypertension and obesity, 2) restricted access to preventive interventions and resources supporting early management of the disease, and 3) limited availability of renal replacement therapy (RRT), primarily among the low-income population [1, 2, 4, 5].

The most recent national health survey (2018) revealed that 10.3% of adults over 20 reported that they had been diagnosed with diabetes by a doctor, 18.4% reported having been diagnosed with arterial hypertension, and 75.2% were overweight or obese [7] -which positions Mexico as one of the countries with the highest rate of overweight and obesity in the world- [8]. These risk factors, taken as a whole, have generated a significant disease burden that translates into years of healthy life lost, mainly due to premature deaths [1, 3–5, 9].

Considering Mexico’s segmented health system, the situation becomes more complex. On the one hand, patients with health insurance (employed, salaried workers) and those with the capacity to pay for private medical care have access to an unrestricted menu of services, including all the RRT options (dialysis, haemodialysis and kidney transplants). On the other hand, patients without health insurance (mainly unsalaried workers, the unemployed and the economically inactive population) have no guarantee of continued dialysis and haemodialysis treatments when they are in the advanced stages of the disease and have very low chances of receiving a kidney transplant [10, 11]. Consequently, patients with end-stage renal disease (ESRD) that do not have health insurance find themselves constantly searching for medical care, and can only access suboptimal treatments via intermittent haemodialysis, either in hospitals that are part of the public hospital network of the Federal Health Secretary or by using their own resources to obtain treatment in private clinics and hospitals [12–16]. As a result, the differences in coverage and access to medical care for ESRD patients have greatly contributed to deepening the health inequities among the Mexican population, with the most unfavourable results occurring among its poorest members.

While various studies have shown that lower-income ESRD patients face more difficulties in accessing medical care than do higher-income patients and that they experience these difficulties more frequently, [4, 17–19], few studies have explored the diverse strategies employed by the uninsured population to address CKD from the first appearance of its signs to obtaining treatment. However, bringing these health care trajectories to light from the perspective of patients and their families is fundamental to adequately orienting medical services and to eliminating the barriers that affect patients’ medical care search, access and quality [20, 21]. For these reasons, the objective of this study is to describe the health care trajectories of patients with ESRD in a public hospital in Mexico City and the barriers they face in receiving RRT (peritoneal dialysis and haemodialysis).

In general, health care trajectories refer to the sum of the actions and interactions between a patient and a health system and its various actors [20]. This trajectory begins at the moment a health care need is identified, and it may contain several phases and involve multiple types of health care providers and institutions before the patient obtains the required treatment and can resolve the health problem [20, 22]. These trajectories are shaped by their own political, sociocultural and economic contexts and by the specific times and places in which they occur, which are influenced by individual, family, community and institutional factors. Trajectories may be affected by multiple social, economic, geographic or organizational barriers, among other types of difficulties [20, 21].

## Methods

### Study design

This study used a convergent mixed methods approach that was predominantly qualitative. The data were collected between September 2016 and February 2017 in the outpatient clinic and the haemodialysis clinic of a public hospital. This institution offers tertiary care in Mexico City and primarily serves people without health insurance.

To improve the range and depth of the study, we combined quantitative and qualitative data. The quantitative data were used to describe and characterize the population and to reconstruct their medical care history, while the qualitative component served to better understand their experience in their search for medical care, access to services, and treatment for their illness [23, 24]. It also allowed for a more robust exploration of the barriers faced by patients and their families throughout the process. In this sense, both the qualitative and quantitative areas of focus contributed important complementary information [23, 24].

The following criteria were used for participant selection. Patients had to meet the following requirements: 1) they had to be 18 or older; 2) they had to be receiving either dialysis or haemodialysis; and 3) they must have a diagnosis of Stage Five CKD according to the *Kidney Disease Improving Global Outcomes Guidelines* (KDIGO) [25] (this information was provided directly by the hospital’s nephrology services staff). Family members had to meet the following requirements: 1) they had to be 18 or older; 2) they had to be the patient’s permanent caregiver; and 3) they had to understand the patient’s medical history. All participants, both patients and family members, must have voluntarily agreed to participate in the study. Participants were contacted directly in the waiting rooms of the facility where, after a presentation about the study’s objectives and the signing of an informed consent form, they were invited to answer the survey questions. The interviews were conducted before or after the patients’ peritoneal dialysis or haemodialysis sessions.

### Data collection and analysis

#### Quantitative component

##### Participants

Over a period of 3 months, 218 surveys were conducted until no new cases satisfying the inclusion criteria could be found. The surveys could be taken by the patients, their family members, or both. Only 199 of the 218 surveys were included in this analysis, as only those with complete and consistent information about the patients’ medical care trajectories were considered.

##### Study variables

The study variables included the following: Demographic characteristics: sex, age, state of residence, type of location (urban, rural), education level (none/elementary, middle school or higher), occupation (none/unemployed/student or self-employed/informal work/agricultural work), and individual or family monthly income (< 110 USD, 110–330 USD, or > 330 USD); health conditions: self-perception of health (very bad/bad, fair, or very good/good), self-reporting of diabetes and/or hypertension (yes or no), limitations on activities of daily living (walking, bathing, or going to the bathroom) (yes or no), difficulties with activities of daily living (doing less than desired or finding difficulty in daily activities because of the presence of CKD within the past month) (yes or no); and treatment history: reasons for beginning CKD treatment, first RRT treatment received (pre-dialysis, peritoneal dialysis, or haemodialysis), current RRT (peritoneal dialysis, or haemodialysis), time that elapsed between the first prescription for treatment and the beginning of treatment, time spent in RRT, frequency of RRT (reaching the suitable level of at least three haemodialysis sessions per week) (yes or no).

##### Procedures

Three trained, standardized surveyors administered a structured questionnaire regarding the study variables, with an average duration of 30 min. Before the fieldwork, a pilot study was conducted with 15 patients to evaluate the questionnaire, measure the time required to complete it, and adjust the wording of the questions.

##### Analysis

We used averages for the description of the variables (using standard deviation [SD]) and applied frequencies and percentages to the categorical variables. The data were analysed using the programme *Statistical Package for the Social Sciences*, version 15.0®.

#### Qualitative component

##### Participants

Interviewers conducted 42 interviews with patients and family members, none of whom were part of the quantitative study. Family members were interviewed when patients could not participate or did not want to participate directly in the study for various reasons (e.g., health conditions that prevented them from answering the questions, not knowing the answers, not having time). In total, 21 interviews were conducted with patients and 21 were conducted with family members. The sample was selected using convenience sampling until the point of category saturation was reached; that is, until new properties or features related to our areas of interest no longer emerged with the addition of new data [26].

##### Procedures

The qualitative component consisted of semi-structured interviews, for which we developed a guide (Additional file 1). The topics treated in the interviews included the patients’ history with the disease, their description of their search for medical attention, their treatment experiences, the barriers they faced in obtaining care and treatment, their perception of the health services and healthcare professionals, their social support networks, and their unsatisfied needs. The interviews with patients and family members were conducted by two health professionals with experience using this interview technique who always maintained the confidentiality of the interview. Interviews were conducted in a private location inside the hospital. Audio recordings of the interviews were made with the express consent of the participants, and they were later transcribed *verbatim* in Microsoft Word. At the end of each interview, relevant notes were taken in field logs to complement the analysis. The average duration of the interviews was 40 min. We used numerical codes to identify interviewees, thus the participants remained anonymous throughout the study. Once the relevant quotations were extracted, they were translated into English by researchers involved in this study (MAB, MRR and RVO). Following that, a specialized, native English-speaking translator was contracted to review and correct these translations while preserving the original meanings of the quotations.

##### Analysis

The qualitative data were analysed within the theoretical framework of phenomenology. From this perspective, the participants’ subjective experiences and understandings related to the disease are prioritized [27]. The interviews were processed using ATLAS.ti® 1.6.0 software and analysed by three researchers (MAB, LGR and MCGR) as follows. First, each of the qualitative analysis experts reviewed the transcripts several times to identify the concepts, data, properties and dimensions present in the narratives (using open coding). In the second phase, the researchers established analytical categories and subcategories based on an understanding of the relationships and interactions that had been identified in patients’ health care trajectories as well as the barriers to RRT that had been identified (using axial coding). Finally, the researchers set up central categories, which were created using specific codes drawn from the transcripts to give meaning and context to the participants’ experiences and histories (selective coding) [26]. Throughout this process, an iterative data analysis was conducted using deductive and inductive techniques. All the results were discussed among the research team.

## Results

### Quantitative analysis

Table 1 shows the participants’ demographic and health characteristics. Approximately 53% were men, and the average age of the participants was 42.9 (SD 15.4). Half of the participants were from the State of Mexico (contiguous to Mexico City). Almost half had no education or only an elementary school education, 84.4% did not work or study, and just over half received a low level of income (< 110 USD per month). Approximately four in ten of those surveyed believed that their health was bad or very bad, while 31% believed that it was fair.

**Table 1:** Demographic and health characteristics of patients with ESRD

Characteristics	N (199)	%
**Demographic variables**		
***Age (SD)***	42.9 (15.4)	
***Sex***		
Male	105	52.8
Female	94	47.2
***State of residence***		
Mexico City	81	40.7
State of Mexico	108	54.3
Other states	10	5.0
***Type of location***		
Urban	172	86.4
Rural	17	8.5
DK/NR (Doesn’t know/no response)	10	5.0
***Marital status***		
Unmarried/Divorced/Widowed	103	51.8
Married/Cohabitation	96	48.2
***Level of education completed***		
None/Elementary school	86	43.2
Middle school and higher	113	56.8
***Occupation***		
None/Unemployed/Student	168	84.4
Self-employed/Informal work/Agricultural work	31	15.6
**Monthly income**^a^**(individual or family)**		
< 110 USD	104	52.3
110–330 USD	66	33.2
> 330 USD	10	5.0
DK/NR	19	9.5
**Health variables**		
***Self-perception of health***		
Very bad/Bad	77	38.7
Fair	62	31.2
Very good/Good	60	30.2
***Diabetes***		
No	117	58.8
Yes	82	41.2
***Hypertension***		
No	125	62.8
Yes	74	37.2
***Limitations in activities of daily living***		
No	135	67.8
Yes	64	32.2
***Difficulty with activities of daily living in the previous month***		
No	77	38.7
Yes	122	61.3

^a^ Value calculated as of May 2018 using an exchange rate of 20 MXN (Mexican peso) = 1 USD

Describing their health care trajectories, 76.9% of the patients’ first RRT consisted of peritoneal dialysis or haemodialysis. At the time of the survey, 80.4% of participants were undergoing haemodialysis. For most of these patients, a nephrologist had prescribed the treatment that they received, while 14.4% received their prescription from a general physician or an internist. Almost 69% of those surveyed received their first RRT within a month of their prescription. Over 80% had been in treatment for 3 months or longer, 49.7% had been hospitalized at some point in the last year for a CKD-related complication (mainly peritonitis), and 36% said that they had uncertainties about their treatment. Of the haemodialysis patients, 63.8% reported that they were not receiving treatment at an adequate frequency (at least three sessions per week) (Table 2).

**Table 2:** CKD health care trajectories

	N (199)	%
***First treatment received***		
Pre-dialysis	40	20.1
Peritoneal dialysis	61	30.7
Haemodialysis	92	46.2
No response	6	3.0
***Current treatment***		
Peritoneal dialysis	39	19.6
Haemodialysis	160	80.4
***Medical professional who prescribed treatment***		
Nephrologist	171	85.9
Other	28	14.1
***Time elapsed between first prescription and receiving treatment***		
< 31 days	137	68.8
≥ 31 days	62	31.2
***Time spent in renal replacement therapy***		
< 3 months	38	19.1
≥ 3 months	161	80.9
***Continuity of treatment (at least three haemodialysis sessions per week)***		
No	127	63.8
Yes	33	16.6
Does not apply (peritoneal dialysis)	39	19.6
***Hospitalizations for health complications in the last year***		
No	100	50.3
Yes	99	47.9
***Patient has uncertainties regarding treatment***		
No	127	63.8
Yes	72	36.2

### Qualitative analysis

Interviews were conducted with 18 men (12 patients and 6 family members) and 24 women (9 patients and 15 family members). The average age of the patients interviewed was 40.7 (SD 15.8), and the average age of family members was 44.8 (SD 13). Of this total, 16 had no schooling or only an elementary school education. Mexico City was home to 16 of the participants, while 18 came from the State of Mexico and the rest came from other Mexican states. The predominant self-reported employment types were self-employed, informal work or agricultural work.

#### Health care trajectory experiences

Drawing from the interviews, three key phases became clear in the health care trajectories of CKD patients and their families: 1) The identification of the signs and symptoms of the disease; 2) The search for medical attention to treat the disease; and 3) Renal replacement therapy. Table 3 shows some of the most significant selections taken from the interviews related to each of these phases.

**Table 3:** Quotations from interviews with ESRD patients and their family members about their health care trajectories

Subtheme	Quotation (participant number^a^, sex, age of participant)
***Theme 1:****Identification of signs and symptoms*	
Appearance of signs and symptoms	“What happened is that she never showed many signs. Later, she felt exhausted, drained, vomited frequently, was very tired …” (*F6, female, 50).* “I never felt anything in particular, nothing, until I began to feel shivers, extreme cold in my feet, and the need to urinate; until suddenly I stopped urinating. I began to hallucinate from the toxicity. I became delirious.” *(P21, male, 65).* “… it gave me a pain back here, in my waist. It was a severe pain, and I was lying here for a long time. Then, I began to swell and swell …” *(P1, male, 60).*
Interpretation of signs and symptoms	“… 1 day, my cheeks suddenly swelled up completely and my eyelids also began to swell, and my family said it was because I slept so much …” *(P1, male, 60).* “He began to shudder. I thought it was a sore throat or a cold …” *(F16, female, 65).* “… I still let a few months go by because I wasn’t sure what was happening to me. I thought it was depression or something like that. I didn’t treat it as you would a physical disease …” *(P26, male, 51).*
***Theme 2:****The search for medical attention*	
First contact with health services	“I was bringing [my daughter] to various doctors – like four – and they couldn’t figure it out, until we arrived at the emergency room and they told us there that she had Stage 4 chronic kidney failure. They had to give her dialysis because her disease was so far along …” *(F14, female, 52).* “The first time I felt bad they took me to the doctor [mentions the name of a clinic next to a pharmacy] and the doctor just checked my eyes and head and prescribed me some medicine. The medicine didn’t help at all and the next day I felt worse and was vomiting. [My mom] took me back to the doctor and he looked at me again, without taking any tests or anything, and prescribed me some injections that did not help me. On the third day I couldn’t take it anymore and they took me to the emergency room …” *(P27, female, 22).* “I went to the clinic, but they just gave me the runaround. They never sent me to a specialist. I fought hard to get here [the hospital]. We went to lots of hospitals, but they set up a lot of obstacles. There’s little hope that they will see you. We spent 3 months doing that.” *(P21, male, 65).*
Diagnosis	“… the doctor told me that it was gastritis, and prescribed some medicine for that, but later my pain was worse and didn’t go away.” *(P11, female, 20).* “… my joints ached, my elbows and knees. My mom took me to the doctor, and they said it was my thyroid. Later, they took me to the doctor again, and again they said it was my thyroid …” *(P27, female, 22).* “One [doctor] even told her she had cholera. A second doctor said she had appendicitis … but they couldn’t relieve her fever or pain … she remained the same. It kept getting worse, because then the swelling started. In addition, this doctor told her she had appendicitis. Another gave her a pregnancy test because of the possibility that she was pregnant, but it wasn’t that either …” *(F14, female, 52).*
Thoughts and feelings upon receiving diagnosis	“… it’s been terrible for me to feel like my life will be cut short. When they told me that I had chronic renal insufficiency I felt as if I could collapse, because I know that this disease is serious.” *(P26, male, 51).* “[The diagnosis] made me so sad, because I thought, ‘You mean they’re going to put tubes in me?’ and I truly would have rather died. At first, I didn’t want anything; the only thing that I wanted was to die.” *(P17, female, 32).* “… in truth, it didn’t even cross my mind that [chronic kidney disease] might be a serious problem. I thought, ‘it’s bad, but it’ll get better. It’ll be fine …’” *(F13, female, 38).*
***Theme 3:****Renal replacement therapy*	
Emergency medical attention	“The nephrologist [the doctor] started to ask if I knew that my husband could die. He told me that I had to pay for an emergency haemodialysis … because he was very swollen … they gave him the first thirty bags and after that he was hospitalized for about 2 weeks …” *(F3, female, 57).* “I took him to the emergency room and that’s where they detected the Stage 4 chronic renal insufficiency, but by then it was urgent that he receive peritoneal dialysis because the disease was already well advanced. Then they made me sign some papers, and from there to here it’s been a long and difficult journey …” *(F14, female, 52).* “… at the emergency room they told me that my toxins were very high and that I needed dialysis. They explained to me that they needed to put a rigid catheter in my stomach to detoxify my body, because if they didn’t, I would die, because I had so many toxins …” *(P27, female, 22).*
Health complications	“… it turns out [the peritoneal cavity] was infected, because they changed the catheter three times, and the third time it gave him peritonitis … after that, the catheter couldn’t save him …” *(F14, female, 52).* “I arrived here [the Hospital] as a case that ‘could not be helped,’ because I arrived with peritonitis, I arrived swollen up, with the catheter incorrectly inserted. I couldn’t walk, I needed oxygen, I couldn’t move from the bed, I couldn’t turn from one side to another. I was hospitalized for 2 months.” *(P27, female, 22).* “I arrived [to the Hospital] with 5% of my kidney working. I checked myself in, and after that I came every 2 weeks, but I got peritonitis and so I came to the emergency room …” *(P23, male, 30).*

^a^*P* patient; *F* family member

#### Identification of signs and symptoms

Before becoming aware of clear signs for alarm, the participants did not experience pain or discomfort that might have caused serious concern. The first signs of the disease were vomiting, swelling of the lower and upper limbs as well as the face, headaches, lower and upper back pain, fever, chronic fatigue, drowsiness, and burning or bleeding during urination. Patients tended to deemphasise these conditions and confuse them with the common symptoms associated with colds and other minor ailments. They ignored these warnings because they thought that they would be temporary. Patients self-medicated for these symptoms or treated them using alternative methods (massages, teas, compresses, etc.). Some patients’ health conditions deteriorated so much that they experienced states of delirium, loss of consciousness, or loss of mobility, causing them to suspend their daily activities at home, school or work. The persistence or further complications of these initial signs and symptoms eventually led patients to seek medical help, often at the insistence of family members.

#### The search for medical attention

At first, patients went to local health clinics, where private, low-cost medical services are offered (approximately 2–5 USD). Almost all the patients reported that their original diagnosis was incorrect. As a result, interviewees stated that they frequently received prescriptions to treat other illnesses, some of which they never had. They invested time and money to pay for consultations, laboratory exams and transportation, as well as incurring other costs. When they did not improve very much or at all, some patients decided to go to other municipalities or states, which marked the beginning a journey from one healthcare provider to another. On this journey, they were likely to encounter two or more doctors, who sometimes offered differing clinical opinions, before they received their definitive diagnosis.

Typically, the patients would receive a diagnosis after being in critical health for weeks or months, at which time their economic resources had been considerably depleted. Once they were diagnosed with “chronic kidney disease”, the patients reported that they did not know what the disease was or what consequences it had. They did not worry much, since they thought it was something that they could “cure.” Once they understood the true nature of the disease and thus assumed it was a death sentence, the participants reported that they had feelings of fear and desperation. They also reported entering a phase of “denial”, continuing to live their “normal” lives until their health inevitably worsened.

#### Renal replacement therapy

The patients that participated in this study received their diagnoses late, that is, when the disease had already progressed to an advanced stage (stages IV and V). This harsh reality for patients and their families came suddenly, and from there they began a race against time. They had to be admitted to hospitals because of health complications, where their cases were treated urgently. Once hospitalized, they received intermittent peritoneal dialysis or haemodialysis. These are intensive sessions undertaken to eliminate the high levels of toxins in a patient’s blood. For those who lived outside Mexico City, this meant an unexpected journey to the capital, which could be anywhere from four to ten hours away.

The patients’ first renal replacement therapies were described as “terrible” and “painful” moments. A catheter or fistula was inserted into their bodies. The participants reported feeling confused, since they had no idea how long the procedure would take. This emergency treatment lasted days for some patients; others spent weeks or even months in the hospital.

Once they had been stabilized, the patients were informed about the necessity of continuing renal replacement therapy. Only then did they realize the seriousness of their disease and the consequences of ignoring treatment, as well as the economic costs involved. The patients began an intensive search for an opening in a public hospital. Because of the high demand for these services, such spaces can be very hard to find, thus further delaying patients’ access to treatment. While searching for a permanent place to obtain RRT, many patients relapsed, most commonly with peritoneal infections. Thus, they began to cycle through public and private services where they were not admitted as permanent patients but as emergency cases receiving urgent treatments (emergency peritoneal dialysis or haemodialysis), and this cycle sometimes resulted in long hospital stays.

#### Barriers to the treatment of CKD

Barriers to CKD treatment can be classified into three primary groups: 1) barriers to access to treatment, 2) barriers to information and contact with health care providers, and 3) barriers to treatment availability. Table 4 offers an overview of these groups of barriers, illustrating them with selected quotations taken from the interviews with patients and family members.

**Table 4:** Quotations from interviews with ESRD patients and their family members about barriers to treatment

Subtheme	Quotation (participant #^a^, sex, age of participant)
***Theme 1:****Barriers to access to treatment*	
Limited medical coverage	“Personally, I would like the government to support the costs of these expensive diseases. It doesn’t make sense that they support the costs of diseases in which you don’t spend much. One of those diseases, you can pay for, but expensive ones like CKD, well, those you can’t pay for.” *(P8 male, 39).*
Economic effects	“… well, the truth is I don’t have the money for the medicine, only for what they give you here [in the hospital], because the truth is I don’t have money for the erythropoietin, it’s super expensive! Or sometimes the antibiotics that I have to buy for him so that the catheter doesn’t cause an infection, well it’s very difficult to buy those, because I can either pay for the hospitalization or I can pay for the haemodialysis.” *(F14, female, 52).*
	“I work in the fields, and well, that’s work in quotes because just now I’m not working. I’ve set aside my work to be here with her [the patient] and well, this is leading us to ruin, because sooner or later I’m not going to have enough even for transportation.” *(F9, male, 60).*
	“I am a street vendor. I sell little bags of candy to be able to pay for my daughter’s treatment, that’s why I’m here at the hospital—I spend entire days here in the hospital—to complete the procedure to admit her here, to complete the procedure to get her out, to make the payments; for all this you have to be here the whole day, so I don’t have a job. Sometimes it’s very difficult because, well, there’s nowhere to make money …” *(F14, female, 52).*
Interruption of treatment (Discontinuity/Intermittence)	“In fact, lately he’s been very bad, and has swollen up more frequently. They told him two [haemodialysis sessions], but the truth is we just have enough for one. That’s why he’s been so bad, because we haven’t been able to make it; but for now, we don’t have enough for the two [sessions].” *(F13, female, 38).* “… my father is my economic support … I had my three haemodialysis treatments, and I dropped one, because I saw that he really couldn’t [pay for it]. Yesterday the doctors told me that I need that [third session of] haemodialysis, but little by little my body is getting used to two...” *(P17, female, 32).*
***Theme 2:****Barriers to information and contact with health services*	
Wait times and administrative procedures	“I left [Puebla, a state about 140 km or 90 miles from Mexico City] at 4:00 a.m. and I arrived at a quarter to 8:00 a.m. … even then there was a line to get a ticket [for patients to be seen]. The woman [hospital staff] told me to get there earlier, but I can’t come earlier because I don’t have anyone to give me a ride at 3:00 a.m. It’s not because of laziness.” *(F7, female, 54).* “… the main thing I need is faster services so that it doesn’t take so long, and that they simplify the administrative procedures more, because they send you from one place to another and back again and then downstairs. I mean, you’re bouncing around …” *(P4, male, 51).*
Lack of information about the disease	“What is renal insufficiency? I get what it is in general terms, but I wish they would tell me what my daughter has or will have; that I don’t know. We don’t know any of these things … They say that [the disease] is lifelong; but how long is that? How long does a person with renal insufficiency live? How can we fight this disease?” *(F9, male, 60).* “I think we need to research more, to understand this disease more thoroughly, [such as] how to take care of it and prevent lots of things. They should really orient us about what to do and what chronic kidney disease implies …” *(P12, female, 30).*
***Theme 3:****Barriers to treatment availability*	
Insufficient equipment, scarcity of materials, deficient infrastructure	“The care is sometimes good and sometimes it’s bad. Sometimes there’s no machines, or they stop giving haemodialysis because the machines break, I mean, yeah, there are a few of these little details …” *(P12, female, 30).* “… I wish there were more haemodialysis clinics because there are so many people, but there are almost no machines. There aren’t any places [available] … sometimes they don’t give me my medicine because it’s too expensive or because there isn’t any …” *(P27, female, 22)*

^a^*P* patient; *F* family member

#### Barriers to access to treatment

A lack of social security was the first barrier faced by these patients with CKD. As mentioned earlier, patients without health insurance do not have guaranteed access to RRT. From the moment they received their diagnoses, patients faced financial difficulties in covering the many costs of the disease. These include not only direct medical costs (i.e., consultations, medicines, lab tests, catheter or fistula placement, and hospitalizations), but also numerous indirect costs (i.e., transport, lodging and food). These indirect costs apply both to the patient and to their family members. For the patients that lived in other states in Mexico, the situation was even more challenging; many of them were compelled to move to live closer to health services.

This situation was extremely precarious for the patients surveyed. These patients had to abandon their jobs because of their disease and become economically dependent. At the same time, the family members interviewed were also unemployed or performing low-paid, informal labour as domestic workers, labourers, farm workers or street vendors. The patients and their family members frequently recycled bottles and cans or sold candy to make money to cover some of their needs. As another economic strategy, they sought support from other family members, friends, neighbours and nongovernmental organizations, although this support decreased with time. In some cases, participants obtained loans, and some had to mortgage or sell their belongings (property, homes, and cars).

The most immediate consequence caused by this lack of funds was that the patient’s RRT was either intermittent or discontinued. Thus, their therapy became irregular; when patients could obtain it, they obtained the minimum required to survive. The average cost of an RRT session varies, fluctuating between 35 USD in public health institutions and 150 USD in private institutions. For the patients interviewed, this cost was an exorbitant amount relative to their low incomes and had to be factored into the total cost of their health care, including their required medicine, periodic health exams, and possible hospitalizations for several days at a time. Patients reported having received fewer peritoneal dialysis or haemodialysis sessions than they were prescribed by the nephrologist and that they had to prioritize RRT instead of addressing the other complications associated with the disease, such as anaemia.

Not all the patients had geographic barriers to obtaining treatment; most lived in Mexico City and could get from their homes to the health services facilities without serious hardship. However, some patients lived in other states and would travel from two to ten hours to obtain health services. Many arrived early in the morning, between 5:00 and 7:00 a.m., hoping that they would be assigned a hospital entry pass. If they did not obtain one, they would have to wait even longer to be seen or return on a different day.

The interviewees considered the administrative procedures required once inside the institutions to be a burden falling primarily on the person accompanying the patient. However, these bureaucratic procedures affected the patients directly as well, as they were sometimes required to wait hours to receive care while their caregiver completed the extensive paperwork.

#### Barriers to information and contact with health care providers

The interviewees reported that the relationship between the patients and the doctors and nurses providing care was often authoritarian and that the doctors and nurses used complex terminology when explaining the disease. Patients and family members did not always understand these explanations, and this misunderstanding resulted in more questions and confusion. A general lack of information about the disease was a recurrent complaint, as was a lack of information about the medical treatment and patient care that was required at home (diet, sexual activity, abdominal sepsis, etc.). The data that was collected for this study (field log notes) indicated that most of the patients did not fully understand their clinical prognosis, and even fewer understood that the RRT would be permanent. In fact, many participants reported that they had never heard of “chronic kidney disease” until the moment of their diagnosis.

#### Barriers to treatment availability

Additionally, the scarcity of medical resources and personnel specialized in CKD were constant barriers that participants encountered when seeking treatment. Participants repeatedly mentioned that the peritoneal dialysis and haemodialysis machines in public health institutions were in short supply, lengthening their wait times even more. Patients would go from one public hospital to another until they found one that could treat them; only a small number of patients opted to go to private facilities. This was the case even though almost all the patients were receiving emergency treatment at this point, and a number were being treated through “continuous” haemodialysis programmes involving three sessions per week. In addition, participants frequently encountered a lack of necessary materials, which added to their overall cost. Participants protested the lack of enough chairs and beds and the general neglect of the facilities in some hospitals.

## Discussion

As far as we know, this is the first study in Mexico that follows ESRD patients’ health care trajectories from the appearance of the first signs of the disease through their search for medical attention and access to RRT. Using a predominantly qualitative, mixed methods analysis, this study sought to both shed light on the strategies used by patients and their families to resolve their health needs and to understand how patients and their families experience this process, including the barriers they face during every phase of the trajectory.

The results clarify the barriers to treatment that patients face, from the moment that the first signs of their illness appear [28] and show that these barriers intensify once patients begin RRT. In these trajectories, patients and their families do not have clear information about the disease and receive inadequate primary level care. Referral and counter-referral mechanisms are non-existent, and the treatment provided is suboptimal, which has been systematically exposed in the national and international literature [10, 12–15, 29–31]. This situation is further intensified by the low socioeconomic level of the population and the emotional impact of CKD.

Some studies have reported that a low socioeconomic level has an effect on the development, progression and outcome of CKD [4, 9, 10, 17, 18, 31–35]. The poorest people obtain the lowest amount of continuous treatment, have more health complications, are more frequently hospitalized, and experience a disproportionate burden resulting from the disease [9, 10, 17, 33–36]. In addition, they have a higher incidence of mental disorders compared to patients with other chronic illnesses, such as coronary diseases or cancer [37, 38]. Depression, anxiety, sleep disturbances and a feeling of imminent death are constants in patients’ lives [37–39], as evidenced by the patients’ statements in this study.

The combination of the quantitative and qualitative data served to model the objective and subjective factors [24] that had an effect on the patients’ CKD health care trajectories, offering a multidimensional view of this phenomenon. Using the quantitative data, the findings of this study establish that access to continuous health services is one of the greatest difficulties faced by uninsured patients and their families in resolving their medical needs. The qualitative data show that the health care trajectories of these patients are permeated with the opinions, perceptions and understandings that patients construct around the disease. Therefore, new programmes are needed, primarily to prevent CKD, but additionally to intervene in the disease’s initial phases and to prepare patients and families for the imminent demands of future medical care, primarily during the RRT phase [16, 37, 38, 40]. Such programmes can positively affect the behaviour, actions and decisions undertaken by the patients and families facing CKD.

In Mexico, as in other low- and middle-income countries, routine procedures are not sufficient to detect and diagnose CKD, nor do health services have the financial capacity to meet the needs of people with ESRD [18, 19, 28, 29, 31, 34, 36, 41]. This has resulted in a high rate of underdiagnosed and undertreated patients [16, 33]. By examining the health care trajectories of patients with ESRD, we observed that approximately 77% began RRT with peritoneal dialysis or haemodialysis. Only 20% were in pre-dialysis programmes, and almost 70% began their first treatment less than one month after their diagnosis. This outcome implies that the health of many of these patients was already critical when they sought care and that they did not receive an early nephrological referral. Given that CKD progresses slowly, silently and irreversibly and that patients may not know that they have it for years, these findings illustrate the importance of strengthening primary level care and increasing early detection of this disease, particularly for those at high-risk (those who are overweight or obese or have diabetes or hypertension).

Similarly, health literacy campaigns are needed, emphasizing risk factors and self-care [42]. Despite the enormous public health problem that CKD represents in Mexico, there are currently no programmes or preventive campaigns that warn of the risks and consequences of this disease. Additionally, there is no national registry of patients with CKD, which could reveal the prevalence, incidence and outcomes of the disease [10, 16].

Out of all the haemodialysis patients surveyed in this study, 63% were receiving the treatment intermittently, mainly due to economic difficulties. However, in one recent study carried out in the hospital where this research was performed, patients gave other reasons for this phenomenon, such as difficulties learning about and managing peritoneal dialysis, an ignorance of other types of RRT, being satisfied with intermittent treatment, or being on a waiting list for a continuous haemodialysis programme [43]. This research points to several areas of opportunity for health service providers, including improving interpersonal relationships between health professionals, patients and family members; simplifying administrative procedures; and reducing wait times to access care (consultations or treatments) [16]. Likewise, the decentralization of peritoneal dialysis programmes should be promoted to broaden the population that can benefit from these services to those outside the capital cities [10], especially in Mexico’s most marginalized regions.

In line with national and international recommendations, our proposals include the following: a CKD patient registry including all phases of the disease; education and outreach directed at the general population; the creation of new, relevant legislation; promoting organ donation; developing and expanding the health care infrastructure; training general practitioners and nephrologists; creating multidisciplinary CKD care groups (doctors, nurses, psychologists, social workers, and nutritionists); establishing referral and counter-referral systems; and broadening RRT availability among the poorest sectors of the population [10, 16, 17, 28, 33, 44].

Finally, the issues surrounding CKD must be placed on the national agenda, since Mexico has one of the world’s highest CKD mortality rates (ranked sixth in 2017) [1] and this rate has doubled in the last 28 years. Projections indicate that if nothing is done, this rate will continue to increase, particularly among the poorest of the population [33, 45], and will threaten the country’s important Sustainable Development Goal of reducing premature deaths from non-communicable diseases (including CKD) by one-third by 2030 [46, 47]. Despite these projections, it has been documented that CKD is both one of the least-treated chronic diseases in the country and evidences some of its greatest inequalities [46].

### Limitations

While this study produced relevant findings, some limitations should be considered. The study was conducted in one hospital in Mexico City, so its results cannot be generalized to other patients elsewhere in the country. However, as the hospital serves people from many states, it was possible to capture some of the heterogeneity of ESRD patients and their various experiences. Another limitation is the selection bias in the population surveyed, since only patients who sought medical attention were at the hospital. It may be that other people with CKD who have never received a diagnosis and do not have access to RRT may experience additional barriers that were not made visible in this study. These patients cannot be identified by studies like this one; another type of analysis would be needed to account for their experiences. An additional limitation is that our analysis focused only on patients without health insurance, so it is not possible to compare their trajectories and the barriers that they faced with those of patients who do have health insurance. A study that considers both groups would be necessary to more precisely identify the inequalities between these two groups. Finally, another potential limitation is that the information gathered did not distinguish between the type of treatment that patients received, whether it was peritoneal dialysis or haemodialysis. While this was not an objective of the study, it is recommended that subsequent studies address the distinctions in the barriers faced by each treatment group.

## Conclusion

The health care trajectories of the CKD patients examined in this study were characterized by late diagnoses, late treatment, and intermittent medical care and occurred in contexts of economic instability. Both objective and subjective factors influence these trajectories, in which health services interact with patients’ perceptions and understandings around CKD. This outcome implies that addressing CKD will require the integration of a biopsychosocial approach in which sociocultural and health service structures are taken into consideration. Mexico urgently requires the implementation of public policies aimed primarily at disease prevention but also at slowing its progression, reducing its complications and providing funding for the uninsured population in need of RRT. These policies must be based on the perspectives of human rights and equality, and the perspectives of patients, their families and the general population should be included in the policy creation process. Any strategy, however, must be realistically aligned with Mexico’s available human resources, finances and infrastructure.

## Supplementary information

**Additional file 1.** Interview guide.

## Data Availability

All data used and/or analyzed during the current study are available from the corresponding author upon reasonable request.

## Abbreviations

CKD: Chronic kidney disease
ESRD: End stage renal disease
KDIGO: Kidney Disease Improving Global Outcomes Guidelines
MXN: Mexican peso
RRT: Renal replacement therapy
SD: Standard deviation
USD: United States dollar

## References

[1] Bikbov B, Purcell C, Levey AS, Smith M, Abdoli A, Abebe M, Adebayo OM, Afarideh M, Agarwal SK, Agudelo-Botero M (2020). Global, regional, and national burden of chronic kidney disease, 1990-2017: a systematic analysis for the global burden of disease study 2017. Lancet.

[2] Gonzalez-Bedat M, Rosa-Diez G, Pecoits-Filho R, Ferreiro A, Garcia-Garcia G, Cusumano A (2015). Burden of disease: prevalence and incidence of ESRD in Latin America. Clin Nephrol.

[3] Jager KJ, Fraser SDS (2017). The ascending rank of chronic kidney disease in the global burden of disease study. Nephrol Dial Transplant.

[4] Jha V, Garcia-Garcia G, Iseki K, Li Z, Naicker S, Plattner B (2013). Chronic kidney disease: global dimension and perspectives. Lancet..

[5] Agudelo-Botero M, Valdez-Ortiz R, Giraldo-Rodríguez L, González-Robledo MC, Mino-León D, Rosales-Herrera MF (2020). Overview of the burden of chronic kidney disease in Mexico: secondary data analysis based on the global burden of diseases study 2017. BMJ Open.

[6] Institute for Health Metrics and Evaluation. GBD Compare Data Visualization. http://vizhub.healthdata.org/gbd-compare. Accessed 23 Jul 2019.

[7] National Institute of Public Health of Mexico. National Health and Nutrition Survey 2018. Results Report of the National Health and Nutrition Survey. 2018. https://ensanut.insp.mx/encuestas/ensanut2018/doctos/informes/ensanut_2018_presentacion_resultados.pdf. Accessed 12 Feb 2020.

[8] Bello-Chavolla OY, Rojas-Martinez R, Aguilar-Salinas CA, Hernandez-Avila M (2017). Epidemiology of diabetes mellitus in Mexico. Nutr Rev.

[9] Levin A, Tonelli M, Bonventre J, Coresh J, Donner JA, Fogo AB, Fox CS, Gansevoort RT, Heerspink HJL, Jardine M (2017). Global kidney health 2017 and beyond: a roadmap for closing gaps in care, research, and policy. Lancet..

[10] Garcia-Garcia G, Chavez-Iniguez JS (2018). The tragedy of having ESRD in Mexico. Kidney Int Rep.

[11] Gomez Dantes O, Sesma S, Becerril VM, Knaul FM, Arreola H, Frenk J (2011). The health system of Mexico. Salud Publica Mex.

[12] Garcia-Garcia G, Renoirte-Lopez K, Marquez-Magana I (2010). Disparities in renal care in Jalisco, Mexico. Semin Nephrol.

[13] Mercado Martinez FJ, Ramos Herrera IM, Valdez CE (2000). Perspectives of chronically ill patients concerning medical care in Guadalajara, Mexico: a qualitative study. Cad Saude Publica..

[14] Mercado-Martinez FJ, Correa-Mauricio ME (2015). Living in hemodialysis without social insurance: the voices of renal sick people and their families. Salud Publica Mex.

[15] Mercado-Martinez FJ, Hernandez-Ibarra E, Ascencio-Mera CD, Diaz-Medina BA, Padilla-Altamira C, Kierans C (2014). Kidney transplant patients without social protection in health: what do patients say about the economic hardships and impact?. Cad Saude Publica.

[16] National Academy of Medicine of Mexico. Chronic kidney disease in Mexico. Towards a national policy to face it. https://www.anmm.org.mx/publicaciones/ultimas_publicaciones/ENF-RENAL.pdf. Accessed 18 Feb 2020.

[17] Crews DC, Bello AK (2019). Saadi G, world kidney day steering C. burden, access, and disparities in kidney disease. Kidney Int.

[18] Garcia-Garcia G, Jha V, Tao Li PK, Garcia-Garcia G, Couser WG, Erk T (2015). Chronic kidney disease (CKD) in disadvantaged populations. Clin Kidney.

[19] Stanifer JW, Von Isenburg M, Chertow GM, Anand S (2018). Chronic kidney disease care models in low- and middle-income countries: a systematic review. BMJ Glob Health.

[20] Defossez G, Rollet A, Dameron O, Ingrand P (2014). Temporal representation of care trajectories of cancer patients using data from a regional information system: an application in breast cancer. BMC Med Inform Decis Mak.

[21] Hirmas Adauy M, Poffald Angulo L, Jasmen Sepulveda AM, Aguilera Sanhueza X, Delgado Becerra I, Vega MJ (2013). Health care access barriers and facilitators: a qualitative systematic review. Rev Panam Salud Publica.

[22] Donabedian A (1992). The role of outcomes in quality assessment and assurance. QRB Qual Rev Bull.

[23] Jason LA, Reed J (2015). The use of mixed methods in studying a chronic illness. Health Psychol Behav Med.

[24] Tariq S, Woodman J (2013). Using mixed methods in health research. JRSM Short Rep.

[25] KDIGO 2017 Clinical Practice Guideline Update for the Diagnosis, Evaluation, Prevention, and Treatment of Chronic Kidney Disease–Mineral and Bone Disorder (CKD-MBD). https://kdigo.org/wp-content/uploads/2017/02/2017-KDIGO-CKD-MBD-GL-Update.pdf. Accessed 12 Dec 2019.

[26] Strauss A, Corbin J (1998). Basics of qualitative research: techniques and procedures for developing grounded theory.

[27] Davidsen AS (2013). Phenomenological approaches in psychology and health sciences. Qual Res Psychol.

[28] Mejia-Avila RE, Arredondo A, de la Sierra de la Vega LA, Miranda RV, Montano AR. Barriers and facilitators in timely detection of chronic kidney disease: evidences for decision-makers. Arch Med Res. 2020. 10.1016/j.arcmed.2020.04.009. 10.1016/j.arcmed.2020.04.009 32336529

[29] Kierans C, Padilla-Altamira C, Garcia-Garcia G, Ibarra-Hernandez M, Mercado FJ. When health systems are barriers to health care: challenges faced by uninsured Mexican kidney patients. PLoS One. 2013:e54380. 10.1371/journal.pone.0054380 PMC3551810 23349868

[30] Lenz O, Mekala DP, Patel DV, Fornoni A, Metz D, Roth D (2005). Barriers to successful care for chronic kidney disease. BMC Nephrol.

[31] Pereira BJ. Overcoming barriers to the early detection and treatment of chronic kidney disease and improving outcomes for end-stage renal disease. Am J Manag Care. 2002;8:122–35. 11924569

[32] Saran R, Bragg-Gresham JL, Rayner HC, Goodkin DA, Keen ML, Van Dijk PC (2003). Nonadherence in hemodialysis: associations with mortality, hospitalization, and practice patterns in the DOPPS. Kidney Int.

[33] Valdez-Ortiz R, Navarro-Reynoso F, Olvera-Soto MG, Martin-Alemañy G, Rodríguez-Matías A, Hernández-Arciniega CR (2018). Mortality in patients with chronic renal disease without health Insurance in Mexico: opportunities for a National Renal Health Policy. Kidney Int Rep..

[34] Correa-Rotter R (2001). The cost barrier to renal replacement therapy and peritoneal dialysis in the developing world. Perit Dial Int.

[35] Crews DC, Novick TK (2019). Social determinants of CKD hotspots. Semin Nephrol.

[36] Li PK, Garcia-Garcia G, Lui SF, Andreoli S, Fung WW, Hradsky A (2020). Kidney health for everyone everywhere-from prevention to detection and equitable access to care. Kidney Int.

[37] Aggarwal HK, Jain D, Dabas G, Yadav RK (2017). Prevalence of depression, Anxiety and Insomnia in Chronic Kidney Disease Patients and their Co-Relation with the Demographic Variables. Pril (Makedon Akad Nauk Umet Odd Med Nauki).

[38] Goh ZS, Griva K (2018). Anxiety and depression in patients with end-stage renal disease: impact and management challenges - a narrative review. Int J Nephrol Renovasc Dis.

[39] Garcia-Llana H, Remor E, Selgas R (2013). Adherence to treatment, emotional state and quality of life in patients with end-stage renal disease undergoing dialysis. Psicothema..

[40] Filgueiras de Assis Mello MV, Angelo M (2018). The impact of chronic kidney disease:experiences of patients and relatives from the extreme North of Brazil. Invest Educ Enferm.

[41] Jha V, Arici M, Collins AJ, Garcia-Garcia G, Hemmelgarn BR, Jafar TH (2016). Understanding kidney care needs and implementation strategies in low- and middle-income countries: conclusions from a "kidney disease: improving global outcomes" (KDIGO) controversies conference. Kidney Int.

[42] Jain D, Green JA (2016). Health literacy in kidney disease: review of the literature and implications for clinical practice. World J Nephrol.

[43] Cantu-Quintanilla G, Gomez-Guerrero I, Silva-Garcia CG, Valdez-Ortiz R (2017). Intermittent hemodialysis. Salud Publica Mex.

[44] Hole B, Hemmelgarn B, Brown E, Brown M, McCulloch MI, Zuniga C, Andreoli SP, Blake PG, Couchoud C, Cueto-Manzano AM (2020). Supportive care for end-stage kidney disease: an integral part of kidney services across a range of income settings around the world. Kidney Int Suppl.

[45] Franco-Marina F, Tirado-Gomez LL, Estrada AV, Moreno-Lopez JA, Pacheco-Dominguez RL, Duran-Arenas L, Lopez-Cervantes M (2011). An indirect estimation of current and future inequalities in the frequency of end stage renal disease in Mexico. Salud Publica Mex.

[46] Luyckx VA, Tonelli M, Stanifer JW (2018). The global burden of kidney disease and the sustainable development goals. Bull World Health Organ.

[47] United Nations Development Programme. Sustainable Development Goals. http://www.who.int/topics/sustainable-development-goals/targets/en/. Accessed 10 Dec 2019.

