# Supplementary material for: Health care trajectories and barriers to treatment for patients with end-stage renal disease without health insurance in Mexico: a mixed methods approach
Source: Int J Equity Health. 2020 Jun 8;19:90. doi: 10.1186/s12939-020-01205-4 (PMC7282114; doi:10.1186/s12939-020-01205-4)
Supplement: Supplementary file 1 — Additional file 1. Interview guide. [file 12939_2020_1205_MOESM1_ESM.docx]

**Appendix 1.** Interview guide

***Topic 1: Disease history***

- When did this disease begin for you?
- What problems did you have?
- What was your response to these problems?
- What did you think about what happened?
- At one point did you start to worry?

***Topic 2: The search for medical attention***

- When and why did you decide to go see a doctor?
- Where did you go, and who did you see?
- What diagnosis did you receive?
- How many places did you go to before receiving the CKD diagnosis? How many doctors did you see?
- What did you feel when you received the diagnosis?
- What information did you already have about CKD?
- What medical recommendations did you receive when you were diagnosed?
- What information did they give you about CKD?
- What did they tell you that you should do?

***Topic 3: Renal replacement therapy***

- Where did you receive your first RRT? Who recommended it to you? What treatment did you receive?
- How many hospitals did you have to go to before receiving the treatment? Why?
- What challenges did you face to be able to get the treatment?
- Describe the first time that you received RRT.

***Topic 4: Barriers to treatment***

- What are the primary barriers that you face in order to obtain your RRT?
- How has CKD affected your life? What things have changed?
- What needs do you have regarding this disease?
- If you have ever had to suspend RRT, mention the reasons.
- What do you think of the health services where you were seen? And the staff?
- What uncertainties do you have about this disease?
